# Physiologically Based Simulations of Deuterated Glucose for Quantifying Cell Turnover in Humans

**DOI:** 10.3389/fimmu.2017.00474

**Published:** 2017-04-25

**Authors:** Julio Lahoz-Beneytez, Stephan Schaller, Derek Macallan, Thomas Eissing, Christoph Niederalt, Becca Asquith

**Affiliations:** ^1^Computational Systems Biology, Bayer AG, Leverkusen, Germany; ^2^Theoretical Immunology Group, Faculty of Medicine, Imperial College London, London, UK; ^3^Institute for Infection and Immunity, St. George’s, University of London, London, UK; ^4^St George’s University Hospitals NHS Foundation Trust, London, UK

**Keywords:** deuterium labeling, T cell kinetics, cell turnover, mathematical modeling, systems biology

## Abstract

*In vivo* [6,6-^2^H_2_]-glucose labeling is a state-of-the-art technique for quantifying cell proliferation and cell disappearance in humans. However, there are discrepancies between estimates of T cell proliferation reported in short (1-day) versus long (7-day) ^2^H_2_-glucose studies and very-long (9-week) ^2^H_2_O studies. It has been suggested that these discrepancies arise from underestimation of true glucose exposure from intermittent blood sampling in the 1-day study. Label availability in glucose studies is normally approximated by a “square pulse” (Sq pulse). Since the body glucose pool is small and turns over rapidly, the availability of labeled glucose can be subject to large fluctuations and the Sq pulse approximation may be very inaccurate. Here, we model the pharmacokinetics of exogenous labeled glucose using a physiologically based pharmacokinetic (PBPK) model to assess the impact of a more complete description of label availability as a function of time on estimates of CD4+ and CD8+ T cell proliferation and disappearance. The model enabled us to predict the exposure to labeled glucose during the fasting and de-labeling phases, to capture the fluctuations of labeled glucose availability caused by the intake of food or high-glucose beverages, and to recalculate the proliferation and death rates of immune cells. The PBPK model was used to reanalyze experimental data from three previously published studies using different labeling protocols. Although using the PBPK enrichment profile decreased the 1-day proliferation estimates by about 4 and 7% for CD4 and CD8+ T cells, respectively, differences with the 7-day and 9-week studies remained significant. We conclude that the approximations underlying the “square pulse” approach—recently suggested as the most plausible hypothesis—only explain a component of the discrepancy in published T cell proliferation rate estimates.

## Introduction

Reliable estimates of lymphocyte turnover are important to understand the immune response in health and disease. Until recently, concerns about the potential toxicity of labels, such as BrdU (1), [^3^H]-thymidine (2), or CFSE (3), restricted labeling experiments to *in vitro* and animal studies (4–6). This problem was overcome with the introduction of stable isotope-labeling techniques (7, 8), which have the advantage of being non-toxic at tracer doses (9) and are suitable for use in humans. Both [6,6-^2^H_2_]-glucose (^2^H_2_-glucose) (7) and heavy water (^2^H_2_O) (8) have been used to measure *in vivo* cell turnover. Glucose enters nucleoside biosynthesis through the pentose phosphate pathway, losing one carbon (C1) to form the pentose ring on which purine and pyrimidine nucleosides are subsequently synthesized (7); heavy water contributes deuterium moieties in place of hydrogen at several sites across the nucleoside molecule (10). Labeled nucleotides are then incorporated into newly synthesized DNA, generating labeled DNA. Since both are used at tracer doses, it is assumed that the underlying physiology is not perturbed. Significant human applications of this technique include the study of T cell (11–13), B cell (14, 15), granulocyte (7, 16), NK cell (17), and monocyte (8, 11) dynamics.

**Figure 1:**
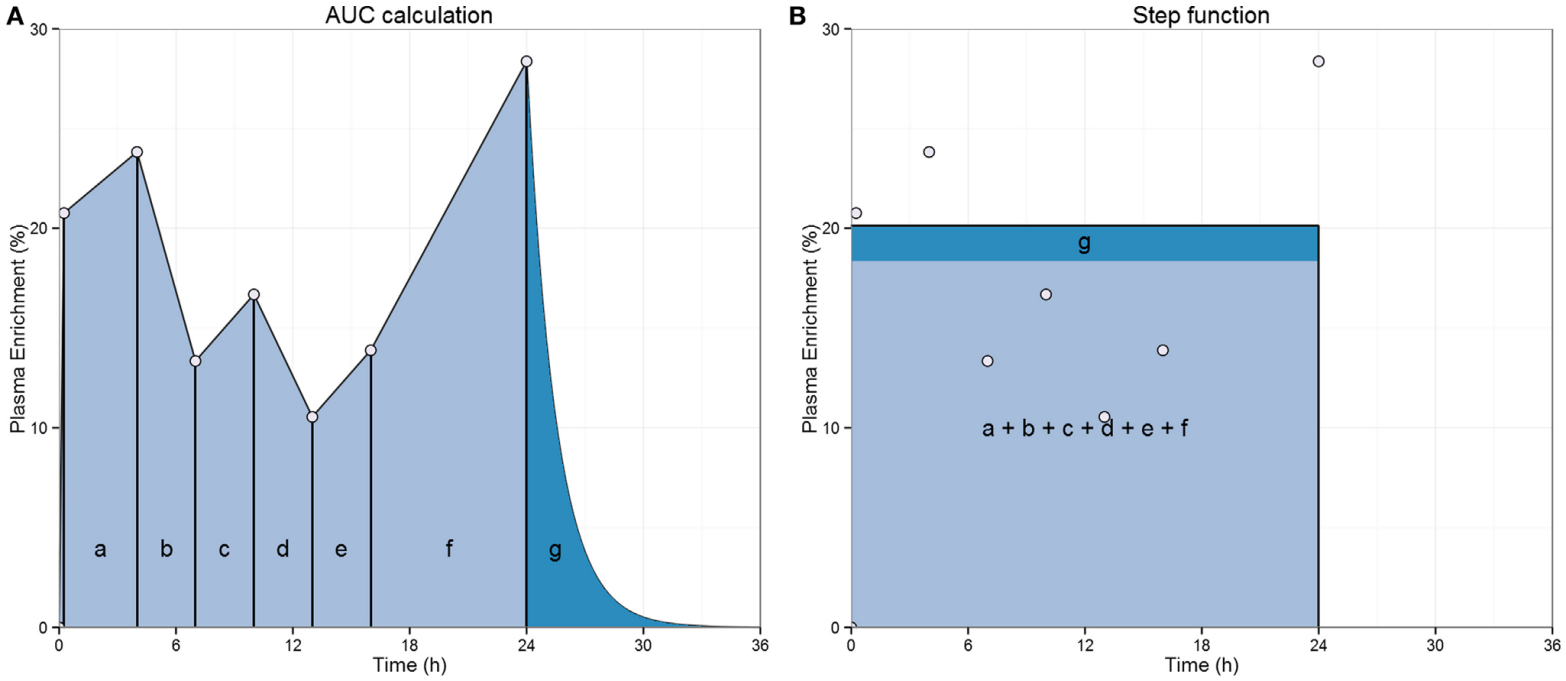
**Illustration of the “square pulse” approach**. **(A)** Label availability during the labeling period in ^2^H_2_-glucose studies is estimated from the area under the curve of plasma label enrichment during the labeling period (typically based on 6–8 measurements). Areas of the trapezoids a–f are summed to obtain the area (light blue shading) representing the average enrichment during the labeling time. An adjustment (dark blue-shaded area, “g”) to account for the de-labeling tail is calculated from the last enrichment data point, glucose pool size, and glucose disappearance to adjust for label availability during the delabelling phase and is added to the mean enrichment (20). **(B)** Label availability is then represented by a “square pulse” whose area is equal to the light blue- plus dark blue-shaded area during the labeling period, which returns to 0 once label administration ends.

Despite the success of this approach, there are considerable discrepancies in the estimates of T cell proliferation reported in ^2^H_2_-glucose studies using 1-day labeling compared with 7-day labeling (18, 19). After a series of *in vitro, in vivo*, and *in silico* studies it was suggested that an underestimation of the ^2^H_2_-glucose enrichment in the 1-day labeling study may have caused an overestimation of the proliferation rates and thus explain the discrepancy (19). ^2^H_2_-glucose studies normally describe label availability as an “square pulse” (Sq pulse) (Figure 1) (20). The “square pulse” approximation might be reasonable in the case of primed and long intravenous infusions where carbohydrate (CHO) intake is limited. However, it may break down when protocols allow large CHO intake, which will cause fluctuations in label availability. In addition, an unbalanced distribution of measurements over postprandial and fasting stages could either under- or overestimate the mean exposure to label. Finally, it has been assumed that the “tail” of residual ^2^H_2_-glucose during delabelling can be accounted for by increasing the amount of label exposure during the labeling phase. Although this may adequately reflect the total level of label exposure, it will change the timing of label availability (Figure 1). This limitation will become more important for shorter labeling periods, when the tail makes a proportionately greater contribution and when the glycogenesis rate is increased by meal intake (^2^H_2_-glucose may be accrued in glycogen and released at later time points).

In this study, we implement a physiologically based pharmacokinetic (PBPK) model to simulate glucose labeling experiments for IV protocols by extending an existing model of the glucose–insulin–glucagon regulatory system (21). This model allows using full label enrichment versus time profiles for the interpretation of labeling experiments, i.e., profiles representing the label enrichment fluctuations during meal intake, account for higher enrichments during the night (when samples are normally not taken), and the tail after glucose infusion. We evaluated the performance of our model by *post hoc in silico* predictions of labeled plasma glucose in human studies. Next, we used our PBPK simulations of label availability to recalculate CD4+ and CD8+ T cell proliferation and disappearance (i.e., due to phenotype change, migration to non-measureable tissues, or death) rates from existing studies in order to test whether this recalculation would resolve the disparity in turnover estimates.

## Materials and Methods

### Datasets

Blood plasma ^2^H_2_-glucose enrichment data from three previously published stable isotope-labeling studies were analyzed (11, 12, 22). Label enrichment in the DNA from T cells was also reported in two of the studies [a 1-day ^2^H_2_-glucose (12) and a 7-day ^2^H_2_-glucose (11)] and used for T cell proliferation and disappearance rate estimation. Only data from healthy individuals were used.

#### Tigas et al. Study

In the study of Tigas et al. (22), ^2^H_2_-glucose was administered in combination with [1-^13^C]-glucose to six healthy young men (average age 28 ± 2 years, BMI 23.7 ± 1.2 kg/m^2^). Only mean data were reported (Table S1 in Supplementary Material). Infusion rates were constant (prime dose 5.95 mg/kg; infusion rate 0.1 mg/kg⋅min for both ^2^H_2_-glucose and [1-^13^C]-glucose). Infusion times of ^2^H_2_-glucose were 2.5 h (protocols A and B), 5 h (protocol C), and 14.5 h (protocol D), starting at time 0 h. A co-infusion of [1-^13^C]-glucose of 2.5 h in protocols A, C, and D (co-infusion start times were 0, 2.5, and 12 h, respectively) and 14.5 h in protocol B (starting time–12 h) was administered. An IV bolus of glucagon was administered 3 h after infusions were concluded. Subjects were fasted throughout the experimental period. Plasma enrichment during the last 30 min of the infusions and during the subsequent 4 h was measured. Priming doses, infusion doses, and infusion times are available in Table S2 in Supplementary Material.

#### Macallan et al. (1-Day Labeling) Study

Macallan et al. (12) administered a primed IV infusion of approximately 1 g/kg ^2^H_2_-glucose to eight healthy subjects (four males, four females) over a period of approximately 24 h; exact dose/times varied but were recorded (see Tables S3 and S4 in Supplementary Material for specific anthropometric data and dosing scheme, respectively). Subjects received up to six meals during the infusion time (see Tables S5 and S6 in Supplementary Material for details in meal intakes).

#### Mohri et al. (7-Day Labeling) Study

Mohri et al. (11) administered a primed IV infusion of 30 g/day of labeled glucose for 7 days to four healthy controls (two males, two females). During the labeling period, subjects received a very-low CHO diet (<46 g/day). Anthropometric data, priming dose, and meal intake times were not recorded. Sampling was performed after an overnight fasting.

#### Interpretation of T Cell DNA Labeling Data

In the 1-day ^2^H_2_-glucose study (12), both CD8+ and CD8− CD3+ T cell populations were sorted into naïve (defined as CD45RA+) and memory (CD45RA−) subpopulations before analysis (12). In the 7-day ^2^H_2_-glucose study, total CD4+ and CD8+ CD3+ T cell populations were sorted and analyzed (11). To compare T cell data from the two studies therefore, naïve and memory T cell data from the 1-day ^2^H_2_-glucose study were combined, weighting the enrichment of each subpopulation according to the relative sizes of the CD45RA+ and CD45RA− blood pools, to obtain an enrichment value equivalent to that of a combined total CD8+ and CD8− population. For consistency with prior published studies (12, 19, 23), we refer to CD8− T cells as CD4+ T cells.

### Models

#### ^2^H_2_-Glucose PBPK Model

A PBPK model of the glucose–insulin–glucagon regulatory system recently published by Schaller et al. (21) was extended to account for labeled glucose in addition to unlabeled glucose (Figure S1 in Supplementary Material). A PBPK model is based on an organism-specific parameter set and a compound-specific parameter set (24, 25). The original PBPK model combined three basic PBPK models of glucose, insulin, and glucagon, coupled by pharmacokinetic and pharmacodynamic (PK/PD) interactions: liver glucose metabolism, pancreas insulin/glucagon secretion, fat/muscle GLUT4 insulin-mediated active uptake, and the gut incretin effect. Here, we extend the model to account for the PK/PD processes of labeled glucose in the form of ^2^H_2_-glucose (MW = 182.17 g/mol). We assume (i) PK/PD properties of glucose and ^2^H_2_-glucose are the same—normal glucose and ^2^H_2_-glucose compete for all active transports (i.e., GLUT2, GLUT3, GLUT4, and SGLT-1), cellular metabolism, renal excretion, and glycogen synthesis in a proportion-dependent manner. In addition, the influence of glucose and ^2^H_2_-glucose on hepatic glucose uptake, hepatic glucose production, and insulin and glucagon secretion are additive; (ii) glucose, ^2^H_2_-glucose, glucagon, and insulin PK/PD properties are the same for all individuals; (iii) in muscle and fat cells, glucose is metabolized by glycogenesis and triglyceride synthesis (26), these glucose sinks are implicitly represented in the model by setting the redistribution of glucose from muscle and fat cells back to the interstitial space to 0; (iv) meals are ingested for 10 min, i.e., each meal is modeled as an oral glucose uptake with a duration of 10 min (see Figure S4 in Supplementary Material for further details and impact of assuming different ingestion times); (v) glucose from meals is released instantaneously; (vi) hepatic glycogen (i.e., normal unlabeled glycogen) concentrations are fixed at a mean physiological value [450 mmol/ml wet tissue (27)]; and (vii) label recycling *via* glycogenesis/glycogenolysis is assumed to have an efficiency of 40%, i.e., 40% of the deuterium label is recycled within the glycogenesis/glycogenolysis cycle (see Text S2 in Supplementary Material and Figure S5 in Supplementary Material for further details and impact of assuming different recycling efficiencies). To examine the performance of the ^2^H_2_-glucose PBPK model, we simulated previously published studies that used ^2^H_2_-glucose as a tracer/labeling compound and measured the label enrichment in plasma during labeling phase and/or delabelling phase. The model was implemented using the Computational Systems Biology Software Suite (28) (Bayer AG, Leverkusen, Germany; www.systems-biology.com) version 5.4.2 (PK-Sim 5.4.2 version, MoBi 3.4.2 version). This software is available with version 7.0.0 under the name Open Systems Pharmacology Suite at the GitHub repository www.open-systems-pharmacology.org. ^2^H_2_-glucose model fits were conducted using the MoBi Toolbox for R and the FME package in R statistical software version 2.15.3 (29, 30).

##### Simulations of the Tigas et al. Study

Subject weight, height, sex, ethnicity, and gender were used to generate a PBPK parameterization characteristic of the average adult volunteer of Tigas et al.’s study (Table S1 in Supplementary Material). Since we were not interested in analyzing the [1-^13^C]-glucose enrichment, the [1-^13^C]-glucose was modeled as normal glucose. Protocols A and B had identical ^2^H_2_-glucose infusion rates and time, and results were conflated in the original publication. Protocols A, C, and D were simulated by using the administration times, prime doses, and infusion rates reported by Tigas et al.

##### Simulations for the Macallan et al. (1-Day Labeling) Study

Subject weight, height, sex, ethnicity, and gender were used to generate a PBPK parameterization characteristic of each subject (Table S3 in Supplementary Material). The model was further individualized by including infusion time, prime, and infused dose for each subject reported by Macallan et al. (12) (Table S4 in Supplementary Material). Food intake was extracted from record sheets and the CHO content of each meal estimated either from dietary labeling or published nutritional information (31, 32) (Tables S5 and S6 in Supplementary Material). Since no data were available for food intake after the end of the labeling period, we estimated CHO intake assuming three daily meals for 5 days after the labeling period, together providing a daily CHO intake equivalent to that of the “average” British citizen (237 g/day) (33). CHO intake was modeled as an oral administration of diluted glucose (see model assumptions). Energy content, meal volume, and fraction solid were used to define the meal effects on the gastric emptying time (34, 35). Simulations of the 1-day labeling experiments were optimized by fitting the CHO of meal intake for the individuals for whom a considerable deviation between model predictions of plasma enrichment and experimental data was observed (Figure S2 in Supplementary Material) during postprandial phases. Since there was uncertainty in measurements on both the ordinate (enrichment) and the abscissa (time) axes, orthogonal least squares between simulation and observation were used as the penalty function. The vector of the residual orthogonal distance was calculated as follows (Eq. 1):

(1) $$\text{ROD}=\vec{\text{min}\left(\sqrt{{\left({\vec{y}}_{s}-y_{d\left(n\right)}\right)}^{2}+{\left({\vec{x}}_{s}-x_{s\left(n\right)}\right)}^{2}}\right)}$$

where *n* is the number of observations, *d* subscript stands for data and *s* subscript stands for simulated vector of values. For error normalization, both the abscise and the ordinate axes were standardized by subtracting the mean and by dividing by the SD of the measured times and enrichments, respectively. The best fit CHO content is provided in Table S8 in Supplementary Material.

##### Simulations for the Mohri et al. (7-Day Labeling) Study

A primed IV infusion of 30 g/day of labeled glucose was modeled for 7 days. The prime dose was as assumed to be 6.6% of the daily dose, as in other protocols (12). CHO intake (45 g/day) was split into three different meals (breakfast, lunch, and dinner). Anthropometric data were not available for the study of Mohri et al.; we, therefore, estimated the weight and height by fitting the simulation to the observed plasma enrichment (11). Meal intake after labeling was modeled as in the 1-day ^2^H_2_-glucose experiment.

#### Kinetic Heterogeneity (kh) Model of T Cell Kinetics

The kh model (36) is used to estimate cell proliferation and disappearance from labeling studies. The kh model considers a population of constant size that proliferates at a rate “*p*,” which is representative of the whole population. Labeled cells are lost (due to phenotype change, migration to non-measureable tissues, or death) at a rate “d*” (36), which is representative of the labeled population of cells only.

In order to fit the model to the DNA fractional enrichment, it is necessary to normalize by the maximum fraction of label attainable (65%) in cellular DNA “*b*” (7, 20) and the plasma glucose enrichment (*U_t_*) assuming a rapid equilibration of ^2^H_2_-glucose between plasma, proliferation site, and the intracellular space of T cells. The model is as follows (Eq. 2):

(2) $$\frac{{\mathrm{dA}}^{*}}{\mathrm{dt}}=b\cdot p\cdot A\cdot U_{t}-d^{*}\cdot A^{*}$$

where *A* is the total amount of deoxyadenosine in a cell population and A* is the amount of labeled deoxyadenosine. Equation 2 can be rewritten by dividing by the total deoxyadenosine amount (36), yielding the following:

(3) $$\frac{{\mathrm{dX}}^{*}}{\mathrm{dt}}=b\cdot p\cdot U_{t}-d^{*}\cdot X^{*}$$

where X* is the fraction of labeled deoxyadenosine of cells in the proliferation sites (X* = A*/*A*). This model was used to analyze the datasets from 1-day ^2^H_2_-glucose (12) and 7-day ^2^H_2_-glucose (11) labeling experiments. In the case when we used the glucose PBPK simulations *U_t_* in Eq. 3, above was given by the plasma ^2^H_2_-glucose PBPK simulations shown in the results section. When using the “square pulse” approach, *U_t_* was calculated as described in Figure 1. Finally, for consistency with previous published experiments (11), we included a lag time to represent the delay of cells leaving the proliferation sites (e.g., lymphoid tissue) for the peripheral blood where they are detected, following Mohri et al. (11); this lag time was fixed to half-a-day, we also allowed cell death during the lag time (Eq. 4).

(4) $$L^{*}\left(t\right)=X^{*}\left(t-\mathrm{lag}\right)\cdot e^{-\mathrm{lag}\cdot d^{*}}$$

where L* is the fraction of labeled deoxyadenosine of cells in peripheral blood (the observed variable). Data from each individual were fitted separately using the pseudorandom algorithm in the FME package in R (29, 30, 37).

### Relationship between Administered Dose and Fasting Plasma Enrichment

A linear regression to explain the relationship between infusion rate and plasma enrichment was performed. Only samples taken during fasting periods were considered.

### Label Exposure Required in the Glucose 1-Day Study for Agreement with the Glucose 7-Day and Water 9-Week Labeling Studies

The kh model was fitted to the CD4+ and CD8+ data of each individual simultaneously with the area under the curve (AUC) of the “square pulse” and loss rate as free parameters, while fixing the proliferation rate of each subpopulation to the median values estimated from analyzing the 7-day study dataset.

### Statistical Analyses

Differences were compared using the two-tailed Wilcoxon Signed Rank Sum test for paired data (i.e., when comparing parameter estimates found using two methods of estimating ^2^H_2_-glucose plasma enrichment applied to the same dataset) and the Mann–Whitney test for unpaired data (i.e., when comparing parameter estimates from different datasets) unless otherwise stated. All reported *p* values are uncorrected and two-tailed.

## Results

### ^2^H_2_-Glucose PBPK Model

The ^2^H_2_-glucose PBPK model is an extension of a previously published model of the glucose–insulin–glucagon regulatory system (21). The glucose–insulin–glucagon metabolism model we used consists of four basic PBPK models for the molecules ^2^H_2_-glucose, glucose, insulin, and glucagon, coupled by pharmacodynamic interactions (Figure S1 in Supplementary Material).

#### Model Performace: *Post Hoc* Predictions of a ^2^H_2_-Glucose IV Infusion Protocol

We first assessed model performance by simulating an independent study performed by Tigas et al. (22). *Post hoc* predictions of ^2^H_2_-glucose plasma enrichments were compared with the experimental data. Importantly, no model parameters were fitted at this step.

**Figure 2:**
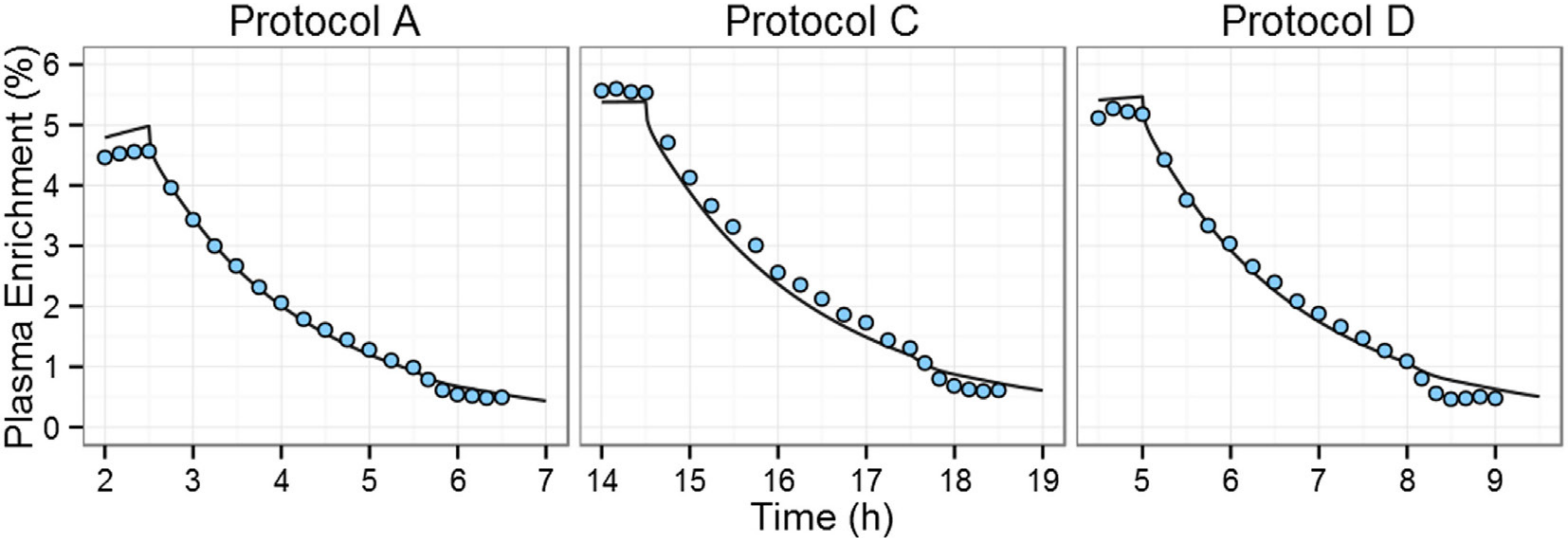
**Comparison of predictions of plasma ^2^H_2_-glucose enrichment to experimental data during and after ^2^H_2_-glucose IV infusion**. Tigas et al. protocols A, C, and D (one graph per protocol) (22). During each protocol, six fasting individuals received both ^2^H_2_-glucose and [1-^13^C]-glucose intravenous infusions for varying times with an injection of glucagon at time +3 h post end-of-infusion. Blue circles represent all the experimental data available in the Tigas et al. publication (data were reported as the mean of six individuals); solid line represents the model prediction.

The ^2^H_2_-glucose enrichments of protocols A, C, and D of the Tigas et al.’s study (22) were simulated using the PBPK model, taking into account the administration of ^2^H_2_-glucose, [1-^13^C]-glucose, and glucagon and using the priming doses, infusion doses, and infusion times reported by Tigas et al. (Table S2 in Supplementary Material). Model predictions described experimental data well (Figure 2), the only exception being that following the glucagon IV bolus the decrease in enrichment in the simulations is smaller than the experimentally observed effect. This is to be expected as the PBPK model was not developed for non-physiological concentrations of glucagon [glucagon plasma concentrations are >2,000 pg/ml after dosing (22)] and cannot, therefore, describe the impact of high-dose exogenous glucagon accurately.

#### Simulation of ^2^H_2_-Glucose Plasma Enrichment during Deuterium Labeling Studies

**Figure 3:**
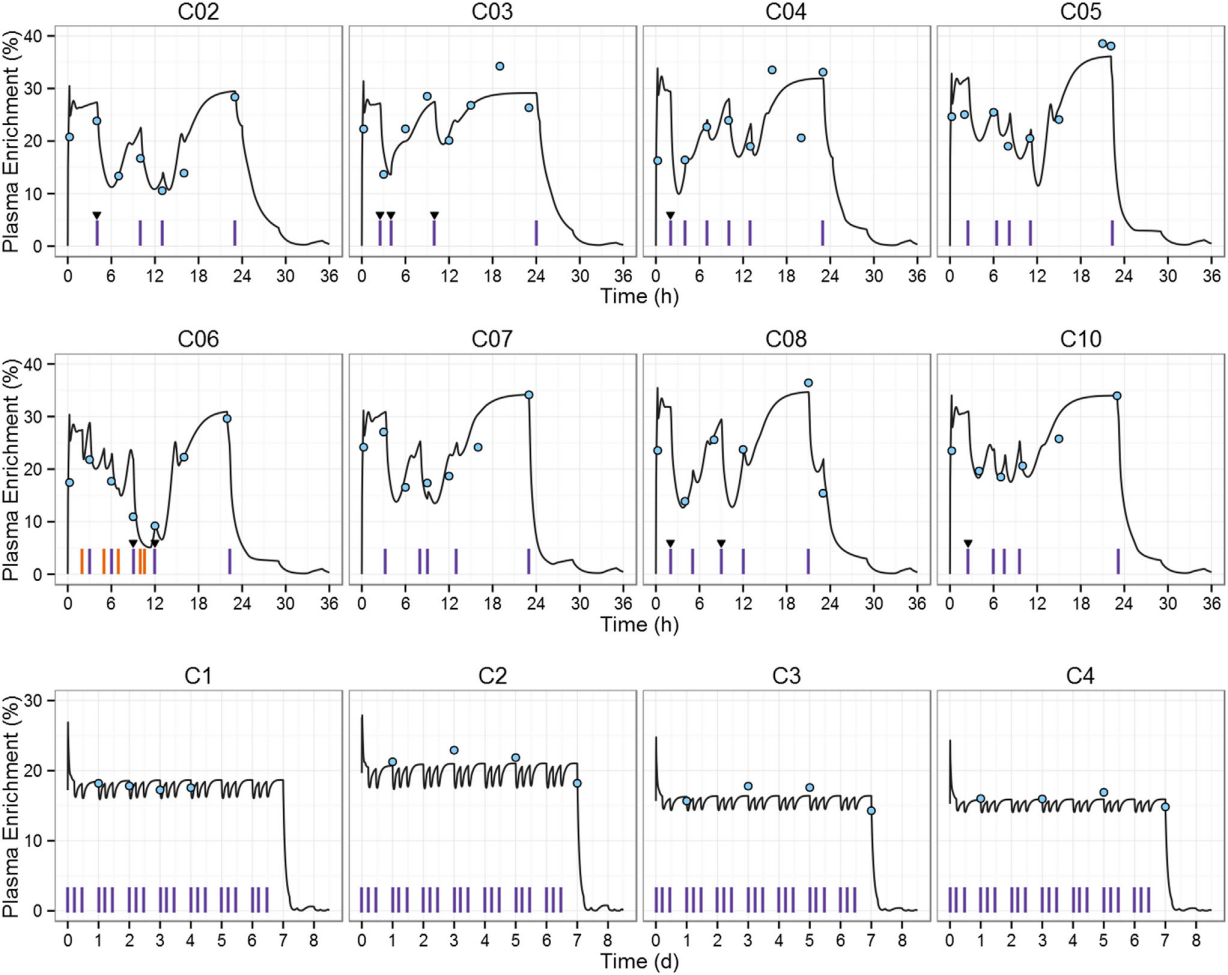
**^2^H_2_-glucose plasma enrichment simulation of the deuterium labeling studies used to estimate T cell proliferation rates**. Macallan et al. (upper eight graphs) dataset (12): each of the eight subjects (C02–C10) received a primed ^2^H_2_-glucose intravenous infusion for approximately 24 h and ingested up to six (unlabeled) meals. Mohri et al. (lower four graphs) dataset (11): each individual (C1–C4) received a ^2^H_2_-glucose primed IV infusion of 30 g/day for 7-day, carbohydrate (CHO) intake was restricted to <46 g/day. Blue circles represent the experimental data, red vertical lines indicate meal intakes during the labeling period, blue vertical lines (C06 plot) represent ingestion of high-glucose beverages (e.g., fruit juice), triangles indicate meals for which CHO content was fitted, and black line represents model prediction.

Next, we simulated the ^2^H_2_-glucose plasma enrichment for the studies that triggered our original research question (11, 12). Macallan et al. (12) administered ^2^H_2_-glucose during 1 day to eight healthy individuals. In contrast to Tigas et al.’s study, individuals were not fasted. Anthropometric characteristics for each individual, priming dose, infusion dose, and infusion times were set to the reported values (see Tables S3 and S4 in Supplementary Material). The PBPK model predictions of ^2^H_2_-glucose enrichment were in good agreement with experimental data during fasting; however, agreement was poorer during the first 4 h of infusion and postabsorptive stages (Figure S2 in Supplementary Material). In order to obtain a better description of the after-meal label fluctuations, the CHO content of meals was fitted in the cases where there was a large discrepancy between prediction and observation as described in the Section “Materials and Methods” (Figure 3, individuals C02–C10; Table S8 in Supplementary Material).

In the study by Mohri et al. (11), a ^2^H_2_-glucose IV primed infusion was administered to four healthy controls for 7 days, label enrichment in plasma was measured after an overnight fast while consuming a very-low CHO diet throughout the labeling study. Anthropometric data were not recorded, so we estimated the weight and height of the four individuals by fitting the simulated fasting enrichment to the data of Mohri et al. (11). Estimates are in the physiological range (Table S9 in Supplementary Material). Results are shown in Figure 3, individuals C1–C4. The mean relative AUC increase compared to the “square pulse” estimation was +9.8% for the 1-day study: 20% (C02), 2% (C03), 1% (C04), 4% (C05), 17% (C06), 13% (C07), 8% (C08), 13% (C10); and −2.5% for the 7-day study: −2 (C1), −4 (C2), −2 (C3), and −2 (C4).

### Analysis of T Cell Stable Isotope-Labeling Data

It has been hypothesized that use of the “square pulse” approach (20) might have led to biased estimates of proliferation rates between the 1-day and 7-day ^2^H_2_-glucose studies described above due to unbalanced sampling between postabsorptive and fasting stages and/or changes in the timing of label availability, i.e., the delabelling tail (19). We aimed to address this question by using PBPK model simulations as an input to a T cell turnover model to recalculate cellular proliferation and death.

**Figure 4:**
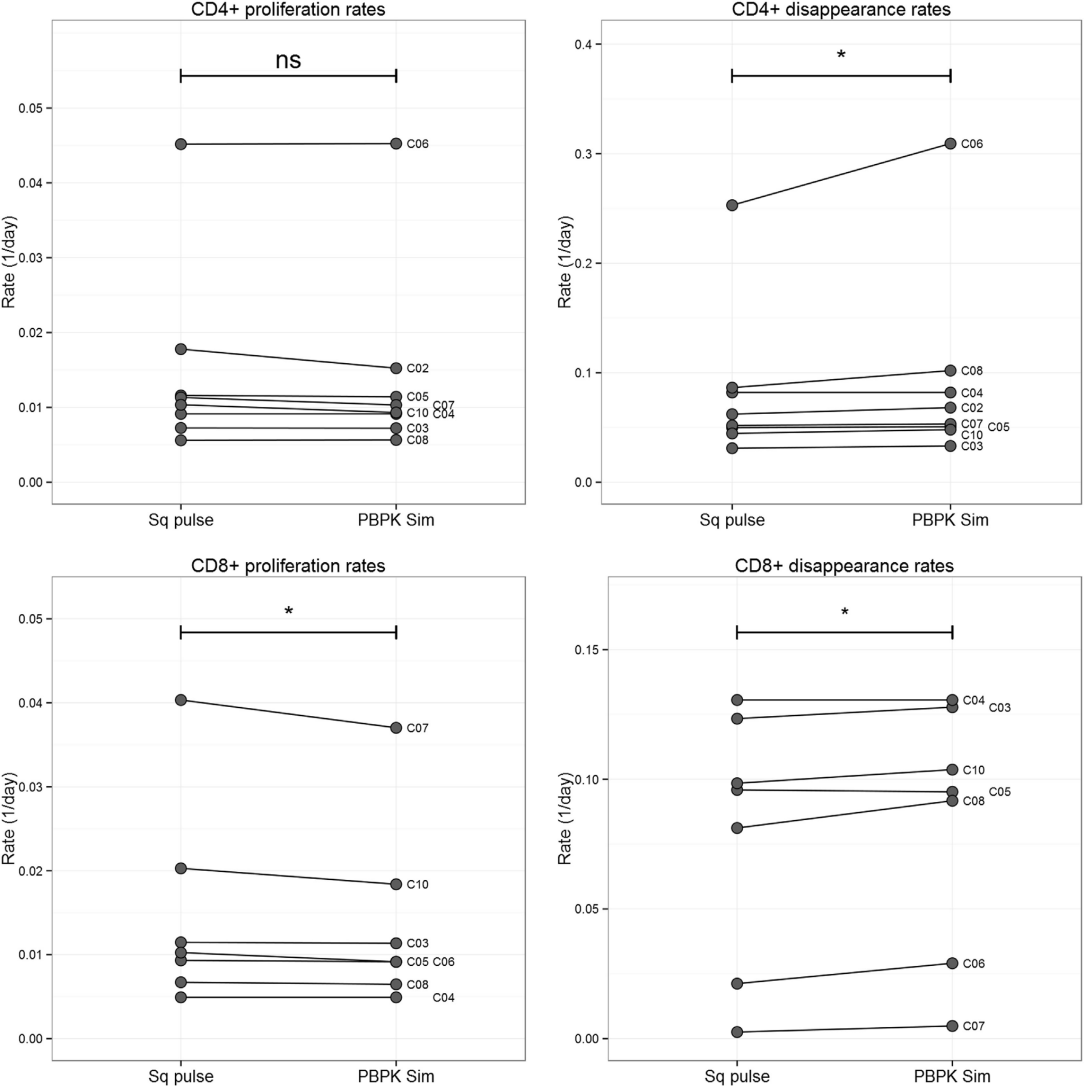
**Comparison T cell proliferation and disappearance rate estimates obtained for the 1-day labeling experiment dataset using “square pulse” and physiologically based pharmacokinetic (PBPK) simulations**. Plasma label enrichment was represented either by a “square pulse” (Sq pulse) or by PBPK model simulation (PBPK Sim). A kinetic heterogeneity (kh) model describing T cell dynamics (36) was then fitted to labeling data from CD4+ and CD8+ T cells in eight individuals labeled with deuterated glucose for 1 day (see Figure S3 in Supplementary Material for model fits). The resulting estimates of the proliferation rate and disappearance rate of the T cell subsets are represented above. Significance levels: *p < 0.05; ^ns^*p* > 0.05, two-tailed Wilcoxon Signed-Rank Sum test.

Comparison of the estimates obtained by the conventional approach (“square pulse”) and the PBPK simulations showed a statistically significant, but numerically small, decrease in the proliferation of CD8+ T cells (mean relative change −7%; *p* < 0.05, two-tailed Wilcoxon Signed-Rank Sum test), but not in CD4+ T cells estimates (Figure 4). On the other hand, disappearance rates significantly increased (mean relative changes 13 and 5% for CD4+ and CD8+ T cell populations, respectively; *p* < 0.05, two-tailed Wilcoxon Signed-Rank Sum test; Figure 4). For the 7-day labeling experiment, plasma ^2^H_2_-glucose PBPK input slightly increased both proliferation rates and disappearance rates of both subpopulations but differences were not significant at the subpopulation level (*p* > 0.12, two-tailed Wilcoxon Signed-Rank Sum test). Model fits are shown in Figure S3 in Supplementary Material.

**Figure 5:**
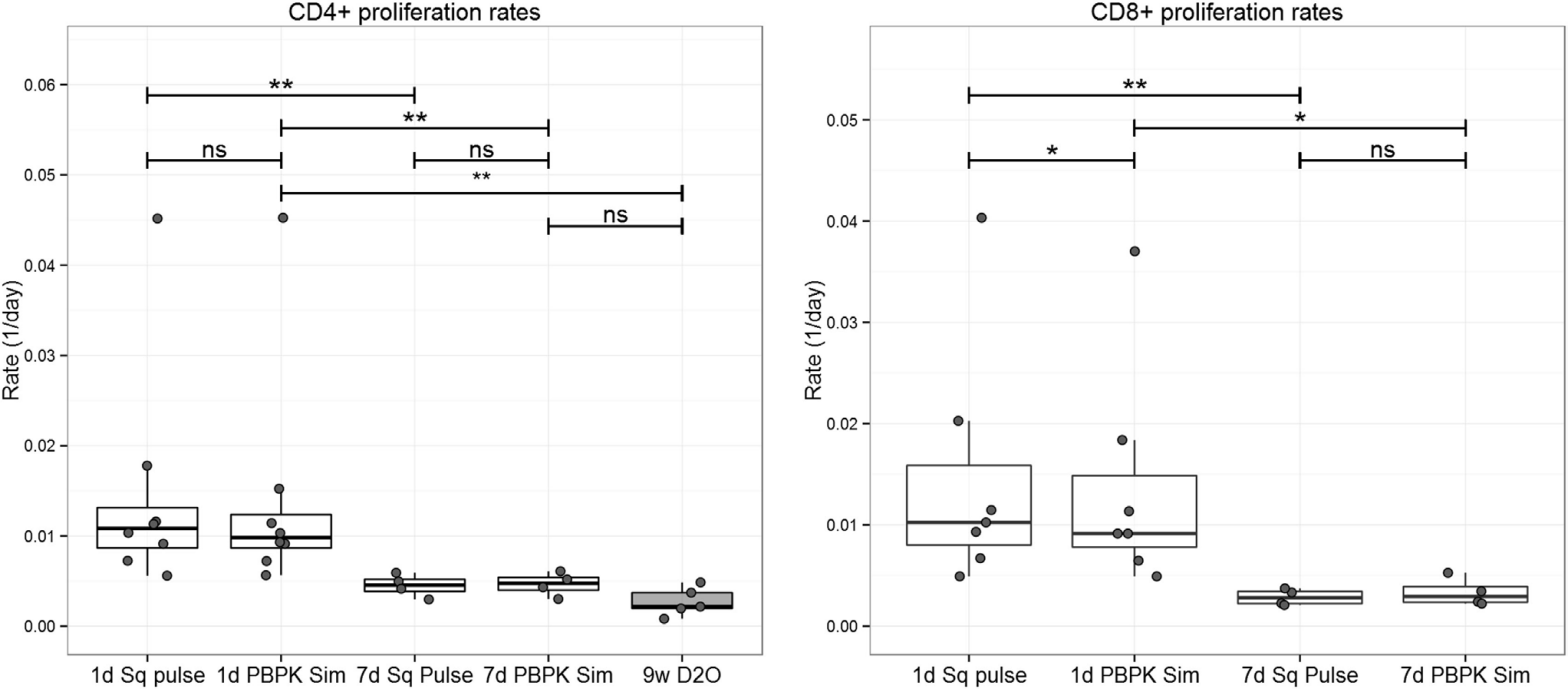
**Comparison of proliferation estimates for the ^2^H_2_-glucose 1-day (12), ^2^H_2_-glucose 7-day (11), and 9-week ^2^H_2_O (23) labeling experiment datasets**. ^2^H_2_-glucose enrichment input for the kh model (36) was represented by the “square pulse” (Sq pulse) or by PBPK model simulation (PBPK Sim). The kh model was then fitted to ^2^H_2_-glucose 1-day and 7-day labeling data from CD4+ and CD8+ T cells (see Figure S3 in Supplementary Material for model fits). CD4+ proliferation rates from the 9-week ^2^H_2_O (D2O) labeling experiment were extracted from Westera et al. (23) and included for comparison; reconstruction for CD8+ cells from the 9-week ^2^H_2_O labeling study was not possible due to sorting methodology, discussed in Ref. (19). Significance levels: **p < 0.01; *p < 0.05; ns, non-significant; two-tailed Mann–Whitney test for unpaired data, two-tailed Wilcoxon Signed-Rank Sum test for paired data. Dots represent individual data, the box-plot represents the median, and inter-quartile range and whiskers represent the maximum and minimum values—excluding outliers.

Despite using a more accurate representation of label availability differences in proliferation rate, estimates between the 1-day and the 7-day ^2^H_2_-glucose studies remained significant (two-tailed Mann–Whitney test, Figure 5). Differences between the ^2^H_2_-glucose 7-day and the ^2^H_2_O 9-week labeling study remained non-significant (19, 23).

### PBPK Model-Free Analysis of Plasma ^2^H_2_-Glucose Enrichment

Another observation that supported the hypothesis that deuterium enrichment in the 1-day study had been underestimated has been reported (19). It was noticed that in the 1-day study, despite administering twice as much glucose as in the 7-day study, the measured plasma enrichments did not differ twofold (19). This was interpreted as a potential underestimation of the precursor availability (19).

**Figure 6:**
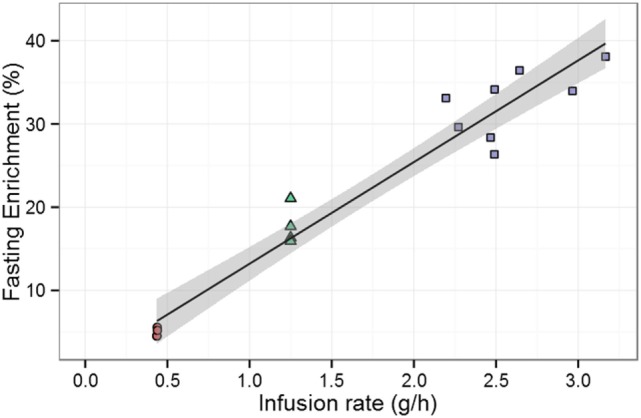
**Effect of infusion rate on experimentally measured enrichment during fasting**. Data are from Tigas et al. (22) (circles), Mohri et al. –7-day labeling– (11) (triangles), and Macallan et al. –1-day labeling– (20) (squares). The solid line is the best fitting straight line through the data; the shaded gray area represents the 95% confidence interval of the mean of the fasting enrichment given a specific infusion rate predicted by the linear model. Pearson product–moment correlation between fasting enrichment and infusion rate is *r* = 0.97, *p* < 0.001 (16 data points).

We investigated this observation by taking into account the nutritional stage of each measurement (i.e., postabsorptive, postprandial, or fasting). In the 7-day study, all measurements were taken after fasting; therefore, in order to be comparable only fasting enrichments from the 1-day labeling study should be considered. On doing this, we found a strong linear correlation between deuterium administration and deuterium enrichment in plasma (Figure 6), consistent with similar rate constants for glucose disposal (Rd)—in the 1-day study, ~8.00 g/h; in the 7-day study, ~7.12 g/h. Bearing in mind the additional 1–2 g/h given in the former, endogenous glucose disposal rates are almost identical, showing that the glucose enrichment estimates are consistent between the two studies. The differences in glucose enrichments may thus be attributed to the differences in meal intake, indicating that the previous conclusion was distorted by not taking into account the differences between postabsorptive and fasting stages in the 1-day study.

### Label Exposure Required in the Glucose 1-Day Study for Agreement with the Glucose 7-Day and Water 9-Week Labeling Studies

Finally, we assessed how much label availability in the 1-day labeling study would need to increase in order to produce T cell turnover rates in agreement with the 7-day study. We found that label availability would need to be three times higher (Table 1) than previously estimated. Such a large error seems unlikely and further supports the conclusion that discrepancies are not entirely caused by the approximations of the “square pulse” approach.

**Table 1:** **Label exposure required (fitted Sq Pulse) in the glucose 1-day study (12) in order to solve the discrepancies with the 7-day (11) and water 9-week (13) labeling studies, compared to original “square pulse” (Sq pulse) exposure and exposure from physiologically based pharmacokinetic simulations (PBPK Sim)**.

	Area under the curve (%·day)		
	Sq pulse	PBPK Sim	fitted Sq Pulse
C02	20.13	24.11	79.55
C03	27.42	28.04	61.85
C04	27.04	27.19	53.84
C05	26.50	27.44	71.94
C06	18.91	22.07	95.68
C07	24.43	27.69	99.99
C08	25.04	26.96	51.92
C10	25.64	28.96	99.99
Mean	24.39	26.56	76.85

## Discussion

Deuterium labeling is an essential technique for quantifying *in vivo* immune cell turnover in health and disease. We hypothesized that discrepancies in published estimates (18, 19) may arise from underestimation of labeled glucose availability when intermittent sampling is combined with discontinuous feeding. Possible mechanisms include: (i) an inappropriate balance of measurements between postprandial and fasting stages, (ii) rapid fluctuations in label availability after meal intake, and/or (iii) inaccuracy in estimating the contribution from the delabelling curve (19). In order to address these issues, we used a PBPK model to estimate glucose label availability throughout the labeling and delabelling period; and then used this description of precursor availability to re-estimate CD4+ and CD8+ T cell proliferation and disappearance rates. The direction of the change in proliferation rates in both the 1-day and the 7-day labeling study is consistent with previous predictions obtained using an independent method (estimation of monocyte plateau DNA enrichment) (19). Our refined estimates of T cell turnover rates suggest a life span of 104 and 109 days for CD4+ and CD8+ T cells, respectively, if estimated from the 1-day labeling study data, compared to 210 and 341 days for CD4+ and CD8+ T cells, respectively, using the 7-day data. The differences between turnover rates estimated using the “square pulse” approach and the PBPK profiles are numerically small (−4 and −7%, for CD4+ and CD8+ data, respectively, 1-day labeling study) and thus only partially resolve the discrepancies in T cell proliferation rate estimates between 1-day and 7-day studies. Interestingly, estimates of the rate of disappearance of CD4+ and CD8+ T cells increased when calculated using the PBPK enrichment profiles, suggesting that previously published rates were underestimates.

This work demonstrates the power of PBPK modeling to integrate known physiological and biological parameters. Using this approach, we were able to include variability in meal intakes and the effect of a delabelling tail for two published ^2^H_2_-glucose labeling studies (a 1-day labeling protocol and a 7-day labeling protocol). There are, however, two main limitations. First, in previous work in type1 Diabetes *mellitus*, individual parameterization of the PBPK model (including fractional glomerular filtration rates of insulin and glucagon, sensitivities to insulin and glucagon, and the catalytic rate constant of SGLT1) was tenable (21). In the present study, by contrast, data were scarce and we, therefore, used parameterization of the model representative of the mean healthy population. Second, the uncertainty regarding meal intake parameters (i.e., CHO content, meal volume, composition, and ingestion time) could have led to the errors during postprandial phases seen in some individuals in the original PBPK predictions (Figure S2 in Supplementary Material), although other factors not included in the model (e.g., glycemic index, meal protein/fat proportion, intra-occasional variability) may also play a role (21). ^2^H_2_-glucose plasma simulations were taken as the input term to estimate cell kinetics using a T cell turnover model that assumed kinetic heterogeneity (36); this is a low parameter approximation of a multi-compartment model which (provided the populations are not saturated) yields the same average proliferation rate as the multi-compartment model without the problem of overfitting. We tested other phenomenological models of T cell turnover and found that differences between T cell turnover estimates from 1 day and 7 day labeling were still significant (Text S1 in Supplementary Material). This suggested that differences are not a particular artifact of the T cell turnover model used.

In summary, the results presented here show that discrepancies are not entirely caused by the approximations of the “square pulse” approach, although it may result in an overestimation of proliferation rates of about 4 and 7% for CD4 and CD8+ T cells, respectively, in short labeling studies. Going forward, difficulties in estimating label availability may be minimized in future studies by limiting the CHO content of meals to reduce label fluctuations, as was done in the 7-day labeling study, and by more frequent plasma sampling during and after label administration, especially during short labeling protocols. Why the 1-day 2H_2_-glucose, 7-day 2H_2_-glucose, and 9-week 2H_2_O studies yield different estimates of T cell turnover is still not completely explained. Alternative explanations might be sought by the application of physiologically based models of T cell proliferation, including trafficking between blood (i.e., sampling site) and non-blood (i.e., proliferating sites) compartments.

## Author Contributions

The contributions of the authors were study conception and design: JL-B, TE, CN, and BA; PBPK model development and simulations: JL-B and SS; T cell turnover modelling and estimates: JL-B and BA; analysis and interpretation of data; critical revision: JL-B, SS, DM, TE, CN, and BA; drafting of manuscript: JL-B, CN, BA, and DM.

## Conflict of Interest Statement

JL-B, SS, TE, and CN are, or were at the time of performing the study, employees of Bayer AG, the company developing PK-Sim and MoBi, and are potential parent company stock owners. DM and BA declare no competing interests.
